# Seasonal Dynamics of ‘*Candidatus* Phytoplasma prunorum’ in Selected *Prunus* Species Revealed by Quantitative PCR

**DOI:** 10.3390/microorganisms14092012

**Published:** 2026-09-10

**Authors:** Peter Morvay, Tomáš Kiss, Ivo Ondrášek, Erika Slámová, Eliška Zezulová, Tomáš Nečas

**Affiliations:** Department of Fruit Science, Faculty of Horticulture, Mendel University in Brno, Zemědělská 1, 613 00 Brno, Czech Republic; peter.morvay@mendelu.cz (P.M.);

**Keywords:** European stone fruit yellows, absolute quantification, phloem colonization, pathogen detection, host–pathogen interaction, stone fruits

## Abstract

The distribution of *‘Candidatus* Phytoplasma prunorum’, a phloem-limited pathogen, varies among plant tissues during the year, but knowledge about quantitative comparisons of its presence in roots and above-ground tissues across *Prunus* species remains limited. The variation in ‘*Ca.* P. prunorum’ in four *Prunus* species was monitored monthly during 2025 using quantitative PCR-based absolute quantification. Roots, one-year-old shoots and annual shoots were sampled monthly from 21 infected trees of *Prunus armeniaca*, *Prunus domestica*, *Prunus persica* and *Prunus salicina*. Phytoplasma titer varied with plant part and sampling month, and differences were also observed among the examined host groups. Because cultivar and rootstock were linked to species, these differences cannot be attributed to species alone. Roots provided the most consistent year-round detection, whereas above-ground tissues showed stronger monthly variation during the studied period. *P. domestica* displayed the most distinct pattern: roots remained frequently positive, while one-year-old shoots and particularly annual shoots had lower detection rates and lower phytoplasma titers. Multilocus genotyping detected ten multilocus profiles, but their uneven distribution among only 21 trees precluded a reliable evaluation of their relationship with phytoplasma titer or symptom expression.

## 1. Introduction

‘*Candidatus* Phytoplasma prunorum’ is associated with European stone fruit yellows (ESFY), one of the most economically relevant phytoplasma-associated diseases of cultivated stone fruits in Europe [1,2]. Phytoplasmas are wall-less, phloem-restricted bacteria [3]. Although they were historically considered unculturable, axenic cultivation of several phytoplasma strains has been achieved under defined conditions [4].

The host range of *‘Ca.* P. prunorum’ includes several species of the genus *Prunus*, including apricot (*Prunus armeniaca* L.), peach (*Prunus persica* (L.) Batsch), Japanese plum (*Prunus salicina* Lindl.), European plum (*Prunus domestica* L.), almond (*Prunus amygdalus* Batsch) and several wild or ornamental *Prunus* species [2,5]. Across European fruit-growing regions, ESFY is particularly important in apricot, Japanese plum and peach, whereas *European plum* is generally regarded as a more tolerant host [6]. Sweet cherry (*Prunus avium* (L.) L.) and sour cherry (*Prunus cerasus* L.) represent specific cases because they have been reported to show low susceptibility or a high level of resistance to ESFY, with weak or absent symptoms and restricted pathogen multiplication in plant tissues [7,8].

The pathogen is transmitted by the psyllid vector *Cacopsylla pruni* and through vegetative propagation, including budding, grafting and other forms of clonal multiplication [2,9]. ESFY symptoms include premature bud break, chlorosis or reddening of leaves, leaf rolling, irregular fruit ripening, fruit drop, decline and, in highly susceptible hosts, tree death [2,10]. Apricot is generally considered one of the most susceptible hosts, whereas *Prunus* species and rootstock combinations can differ substantially in their response to infection [2,8,11].

Previous studies have shown that the detectability of ‘*Ca.* P. prunorum’ changes during the year because phytoplasmas are unevenly distributed among host tissues. The pathogen can persist in roots throughout the year, while its detection in above-ground organs may decrease during unfavorable periods, leading to false-negative diagnostic results [2,7,12]. Moreover, phytoplasma titers can differ strongly among positions within a single shoot [13]. This uneven distribution is important both for diagnostics and for the selection of propagation material.

Earlier year-round studies of ‘*Ca.* P. prunorum’ distribution were based mainly on PCR or DAPI staining [7,12]. These methods provided important information on pathogen presence but did not allow precise identification or quantification of phytoplasma titers. Quantitative PCR enables absolute or relative quantification and, therefore, provides a suitable approach for analyzing seasonal changes in phytoplasma titers in different tissues [14,15]. A specific real-time PCR assay has also been developed for sensitive detection and absolute quantification of ‘*Ca*. P. prunorum’ [16].

Populations of ‘*Ca.* P. prunorum’ are genetically heterogeneous, and multilocus analyses of non-ribosomal markers such as aceF, pnp, imp and secY have revealed geographically structured genotype combinations in European plant and insect-vector populations [17,18]. This diversity is epidemiologically relevant because comparisons of genotypes detected in host plants and *C. pruni* can support the investigation of pathogen circulation and geographic spread.

The objective of this study was to quantify monthly variation in ‘*Ca.* P. prunorum’ in selected *Prunus* species and plant tissues using quantitative PCR. Specifically, we evaluated the effects of host species, sampling month and plant part on phytoplasma titer and detection rate. Multilocus genotyping was used to characterize the genetic diversity of the analyzed strains.

## 2. Materials and Methods

### 2.1. Plant Material and Experimental Site

This study was conducted in the experimental orchard of the Department of Fruit Science, Faculty of Horticulture, Mendel University in Brno, Lednice, Czech Republic (southern Moravia; district Břeclav; 173 m a.s.l.). The locality is characterized by a mean annual temperature of approximately 10.5-11.5 °C and mean annual precipitation of 500–600 mm; the experimental orchard is located in the Lednice area, where modal and carbonate Chernozems developed on loess are predominant [19].

Four *Prunus* species were included: *P. armeniaca*, *P. persica*, *P. salicina* and *P. domestica*. Before the experiment, all trees were tested via quantitative PCR using the species-specific ESFY, PD and AP probes according to Nikolić et al. [15]. For each *Prunus* species, two cultivars were selected and represented by 2 to 3 individual trees, resulting in 21 infected trees used in the experiment (Table 1). The apricot trees were approximately 20 years old, whereas trees of the remaining species were approximately 10 years old. Apricot trees were grafted onto apricot seedling rootstocks (*P. armeniaca*). *P. persica* was grafted onto Adesoto (a clonal selection of *Prunus insititia* L.), *P. salicina* onto Penta (a clonal selection of *P. domestica*), and *P. domestica* onto Penta or St. Julien A (a clonal selection of *P. insititia*). Because rootstock was linked to cultivar and host species, rootstock effects were not interpreted as independent main effects. Because cultivar and rootstock were linked to host species, their effects could not be separated; comparisons among species, therefore, represent comparisons among the examined species–cultivar–rootstock combinations.

### 2.2. Sampling

Sampling was performed monthly during 2025, usually between the 20th and 25th day of each month. The crown of each tree was divided into three sectors, and the sampled branches in each sector were permanently labelled to ensure consistency among sampling dates. Roots and one-year-old shoots were sampled from January to December, whereas annual shoots were sampled from May to December according to shoot development. At each sampling date, three one-year-old shoots (one from each sector), three annual shoots (one from each sector) and one root sample were collected per tree whenever the corresponding tissue was available. Root samples were taken from lateral roots with a diameter of approximately 0.5–1.0 cm at a depth of 0.10–0.40 m. Phloem tissue (0.1–0.2 g) was mechanically scraped from roots and from the basal (first 5 cm) parts of one-year-old shoots and annual shoots and weighed precisely to 1 mg. After collection, plant samples were placed in zip-lock bags and stored at 6 °C for no longer than one week before DNA extraction.

### 2.3. DNA Extraction and PCR Quantification

Total DNA was extracted using a modified CTAB+PVP protocol according to Maixner et al. [20], as described by Kiss et al. [11]. Samples were homogenized using a Homex 6 semi-automatic homogenizer (Bioreba, Reinach, Switzerland), and DNA was resuspended in 100 µL of nuclease-free water (Ambion, Austin, TX, USA). DNA extracts were stored at −20 °C until molecular analysis.

The generic phytoplasma real-time PCR assay described by Christensen et al. [14] was performed using the phytoplasma 16S rDNA forward primer, phytoplasma 16S rDNA reverse primer and phytoplasma 16S rDNA probe. All three oligonucleotides target phytoplasmal *16S rRNA* gene; this assay is generic for phytoplasmas. The 10 µL reaction mixture contained 0.3 µM forward primer, 0.9 µM reverse primer, 0.2 µM TaqMan probe, 1 × TaqMan™ Universal Master Mix II, no UNG (Applied Biosystems™, Foster City, CA, USA), 2 µL template DNA and nuclease-free water. Amplification consisted of polymerase activation at 95 °C for 1 min, followed by 40 cycles of 95 °C for 15 s and 60 °C for 60 s. Fluorescence was recorded in the FAM channel using the CFX96 real-time PCR system (Bio-Rad, USA), and data were processed using CFX Maestro Software version 2.0 (Bio-Rad Laboratories, Hercules, CA, USA). All samples were analyzed in two technical replicates.

Absolute quantification was based on a plasmid standard containing the cloned target PCR product prepared by Generi Biotech (Hradec Králové, Czech Republic). The standard was serially diluted in DNA extracted from a negative apricot sample to generate a calibration curve covering 10^7^ to 10^1^ copies.µL^−1^. Results were calculated as phytoplasma cells.g^−1^ phloem using formula below and then log_10_ transformed.

$$x=\frac{A\times B}{C\times D\times 2}$$

x ‘*Ca*. P. prunorum’ cells per gram of plant material (phloem);
A copy number of amplified *16S rRNA* gene in the reaction obtained from CFX Maestro Software version 2.0 (Bio-Rad Laboratories, Hercules, CA, USA);
B dilution factor of the plant sample and DNA extract during the DNA extraction and qPCR processes;
C weight of the plant sample used for the DNA extraction (g);
D amplification efficiency (E = 97.3%) of the qPCR protocol based on the calibration curve of the standard dilutions obtained from CFX Maestro Software version 2.0 (Bio-Rad Laboratories, Hercules, CA, USA);
2 phytoplasma genome of one phytoplasma cell contains two copies of *16S rRNA* gene [1].

### 2.4. Multilocus Genotyping

One sample per tree was selected for multilocus genotyping. The sample with the highest phytoplasma titer, as determined by quantitative PCR, was used for sequencing, and the resulting multilocus profile was assigned to the corresponding tree.

Four molecular markers for *‘Ca.* P. prunorum’ characterization were analyzed: aceF, pnp, imp and secY. DNA fragments were amplified by nested PCR using the marker-specific primer pairs for *‘Ca.* P. prunorum’ and cycling conditions described by Danet et al. [17]. PCR products were checked on 1% agarose gel by electrophoresis, purified using the NucleoSpin Gel and PCR Clean-up kit (Macherey-Nagel, Düren, Germany) according to the manufacturer’s instructions and Sanger sequenced bidirectionally by Eurofins Genomics (Ebersberg, Germany). Chromatograms were inspected using CLC Genomics Workbench version 25.0.1 (QIAGEN Digital Insights, Aarhus, Denmark). Forward and reverse reads were assembled into consensus sequences and aligned using default alignment parameters in CLC Genomics Workbench version 25.0.1 (QIAGEN Digital Insights, Aarhus, Denmark) with the corresponding aceF, pnp, imp and secY reference sequences and genotype datasets reported by Danet et al. [17] and Dermastia et al. [18], which were retrieved from the NCBI GenBank database. For each tree, the combination of the aceF, pnp, imp and secY genotypes was designated as a four-locus multilocus profile.

### 2.5. Symptom Assessment

During the vegetation period, trees were visually inspected for symptoms associated with ESFY, especially leaf chlorosis, leaf reddening, leaf rolling, premature bud break, gummosis and decline symptoms. Symptom observations were used as descriptive support for the molecular results.

### 2.6. Statistical Analysis

Statistical analyses were performed in R version 4.6.0 using the lme4 version 2.0.1, lmer Test version 3.2.1 and emmeans version 2.0.3 packages. Detection rates were summarized as counts and percentages. Samples with negative detection results were coded as zero for detection summaries, whereas titer analyses included qPCR-positive samples only. Negative results were not assigned a concentration of zero because they represented values below the limit of detection of used qPCR protocol (2 log_10_ copies of *16S rRNA* gene in reaction, Supplementary Table S1) rather than confirmed zero concentrations; the reported mean titers are, therefore, conditional on detection. Negative qPCR results were treated as non-detections in the detection-rate summaries. Because phytoplasmas may be unevenly distributed within plant tissues, a negative result for an individual sample was not interpreted as evidence that the phytoplasma was absent from the entire tree.

Detection probability was analyzed using a binomial generalized linear mixed-effects model with a logit link. Host group, plant part, sampling month and the host group × plant part interaction were included as fixed effects, and tree identity was included as a random intercept. A plant part × month interaction was evaluated using a likelihood-ratio test. Pairwise comparisons of estimated marginal detection probabilities among plant parts within each host group were performed using the emmeans package with Holm adjustment.

Because the same trees were sampled repeatedly across months and plant parts, tree identity was included as a random intercept in all linear mixed-effects models. The combined model was specified as log_10_ titer ~ host species × sampling month × plant part + (1 | tree identity). Plant-part-specific models were specified as log_10_ titer ~ host species × sampling month + (1 | tree identity), and models fitted within individual host groups were specified as log_10_ titer ~ plant part × sampling month + (1 | tree identity). Sampling month was treated as a categorical fixed effect; thus, the tree rather than each monthly tissue observation represented the independent biological unit.

Separate LMMs were fitted for roots, one-year-old shoots and annual shoots to determine how the effects of host species and sampling month differed among plant parts. Fixed effects were evaluated using Type III F-tests (F) within the linear mixed-effects models, with the Satterthwaite approximation used to estimate denominator degrees of freedom. For each plant part, the interaction between host species and sampling month was additionally checked using a likelihood-ratio test (LRT), which compared a model allowing monthly changes during 2025 to differ among species with a simpler model assuming the same monthly pattern for all species.

To compare phytoplasma titers among plant parts within individual host species, all available positive samples were used for monthly comparisons. From January to April, comparisons included roots and one-year-old shoots only, because annual shoots had not yet developed sufficiently for sampling. From May to December, all three plant parts were compared. The overall LMMs were restricted to May–December so that all three plant parts were evaluated over the same period. Plant part, sampling month and their interaction were treated as fixed effects, and tree identity was included as a random intercept. The plant part × month interaction was additionally evaluated using a likelihood-ratio test comparing the full model with an additive model.

Post hoc pairwise comparisons of estimated marginal means were performed using the emmeans package with Tukey adjustment for multiple comparisons. Tukey-adjusted *p* < 0.05 was considered statistically significant. Analyses of annual shoots were restricted to May–December, when this plant part was available. For graphical presentation, arithmetic means and standard deviations (SDs) of log-transformed phytoplasma titers were calculated from positive samples within each host species, plant part and sampling month.

## 3. Results

### 3.1. Overall Detection and Tissue Distribution of ‘Ca. P. prunorum’

Pre-experimental testing yielded positive reactions with the ESFY probe for ‘*Ca*. P. prunorum’ in all 21 trees, whereas the PD and AP probes for ‘*Ca*. P. pyri’ and ‘*Ca*. P. mali’, respectively, yielded negative results in all cases.

The occurrence of *‘Ca.* P. prunorum’ was evaluated throughout the sampling period in four *Prunus* species and three plant parts: roots, one-year-old shoots and annual shoots. The detection rate was highest in roots (241 positive/252 tested samples; 95.6%), followed by one-year-old shoots (660 positive/756 tested samples; 87.3%) and annual shoots (385 positive/503 tested samples; 76.5%). Detection rates were high in *P. salicina* (423 positive/432 tested samples; 97.9%), *P. persica* (347 positive/360 tested samples; 96.4%) and *P. armeniaca* (409 positive/431 tested samples; 94.9%), whereas only 107 of 288 tested *P. domestica* samples were positive (37.2%).

The clearest reduction in detection was observed in the above-ground plant parts of *P. domestica*. Roots remained frequently positive (45 positive/48 tested samples; 93.8%), whereas the detection rate decreased to 39.6% in one-year-old shoots (57 positive/144 tested samples) and 5.2% in annual shoots (5 positive/96 tested samples). By contrast, the other host species maintained high detection rates in roots and above-ground plant parts, particularly in one-year-old shoots.

**Figure 1:**
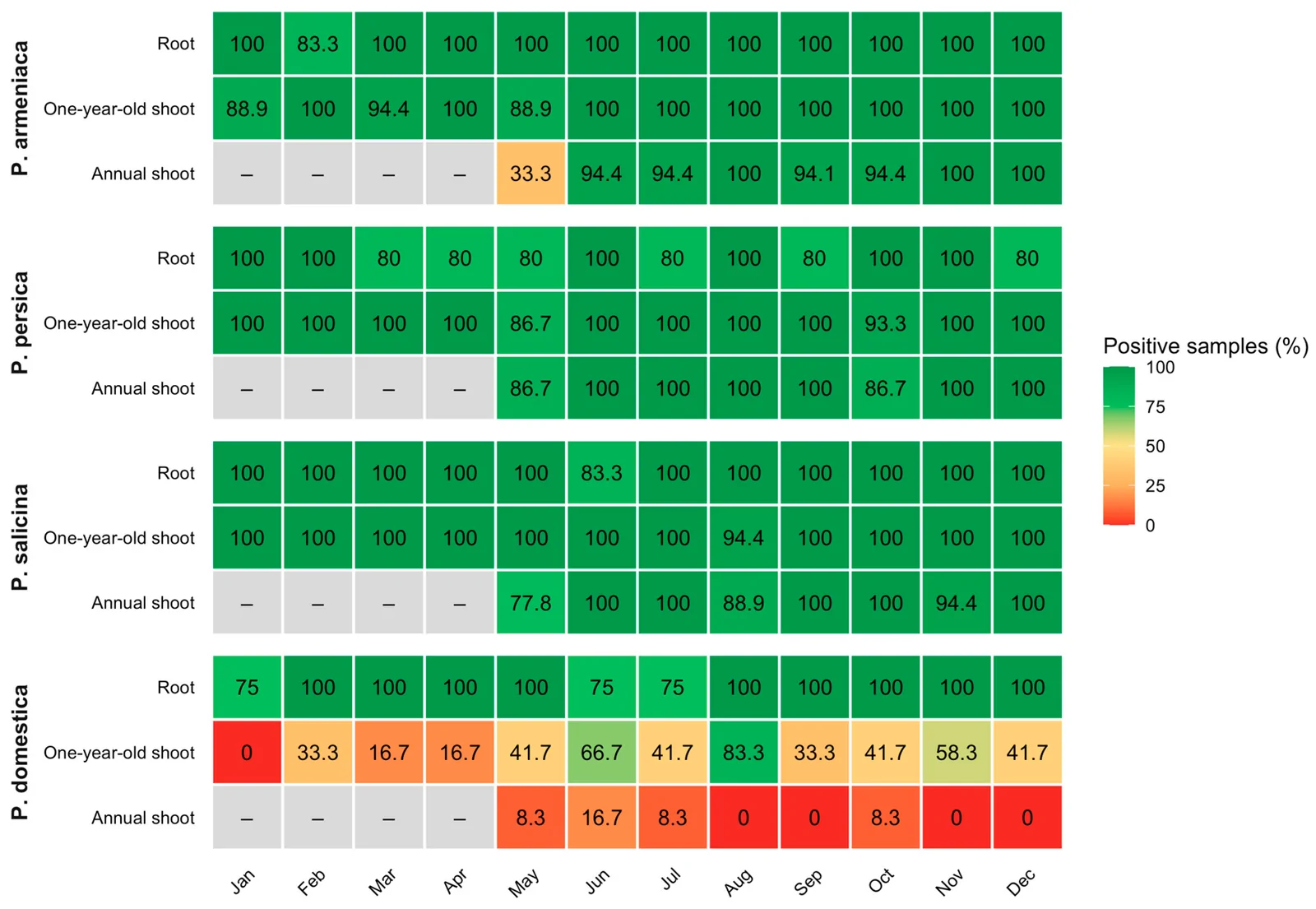
Monthly observed detection rate of ‘*Ca*. P. prunorum’ in three plant parts of four *Prunus* host groups. Values represent the percentages of qPCR-positive samples; gray cells indicate months in which annual shoots were not sampled. Statistical inference was based on the binomial generalized linear mixed-effects model described in Section 2.6.

Monthly observed detection rates are shown in Figure 1. The binomial GLMM detected a significant host group × plant part interaction (likelihood-ratio test: χ^2^ = 58.51, df = 6, *p* < 0.001) and a significant effect of sampling month (χ^2^ = 63.81, df = 11, *p* < 0.001). Adding the plant part × month interaction did not significantly improve the model (χ^2^ = 26.23, df = 18, *p* = 0.095) and was, therefore, not retained. Holm-adjusted post hoc comparisons indicated that detection probability differed among all three plant parts in *P. domestica* (all adjusted *p* < 0.001). In *P. armeniaca*, annual shoots differed from roots (adjusted *p* = 0.025) and one-year-old shoots (adjusted *p* < 0.001). Detection probability also differed between roots and one-year-old shoots in *P. persica* (adjusted *p* = 0.025) and between one-year-old and annual shoots in *P. salicina* (adjusted *p* = 0.035). The remaining within-host comparisons were not significant.

When all positive samples were analyzed together, phytoplasma titers differed significantly among host species (F = 7.96, *p* = 0.001), sampling months (F = 10.95, *p* < 0.001) and plant parts (F = 41.75, *p* < 0.001). These effects were not independent: significant interactions occurred between host species and month (F = 4.89, *p* < 0.001), host species and plant part (F = 45.69, *p* < 0.001), and month and plant part (F = 6.43, *p* < 0.001). The combined interaction among host species, month and plant part was also significant (F = 2.10, *p* < 0.001). In practical terms, differences in phytoplasma titer among roots, one-year-old shoots and annual shoots were not the same in every host species or throughout the year. Roots were comparatively stable, whereas titers in above-ground tissues generally increased during the growing season in *P. armeniaca*, *P. persica* and *P. salicina* but remained low and irregular in *P. domestica*.

### 3.2. Monthly Variation and Distribution of ‘Ca. P. prunorum’ in Plant Parts

When roots, one-year-old shoots and annual shoots were analyzed separately, phytoplasma titer was significantly affected by host species and sampling month, although the magnitude and monthly pattern during 2025 of these effects differed among plant parts (Table 2).

**Figure 2:**
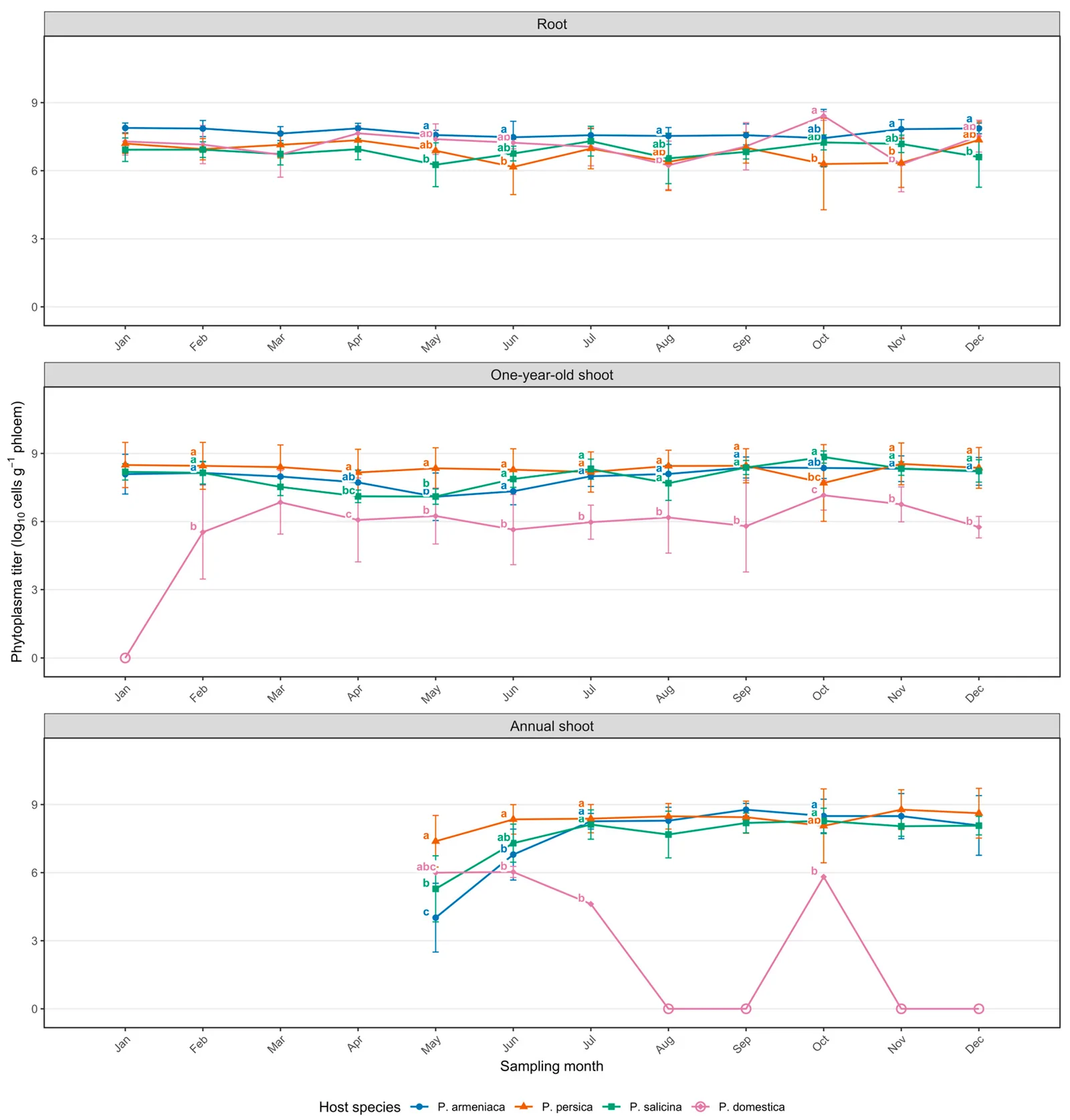
Monthly variation in phytoplasma titer during 2025 in four *Prunus* species within each plant part. Points represent arithmetic means calculated from positive samples, and error bars represent standard deviations (SD). Open points at zero indicate months without positive samples and were excluded from the titer models. Different letters indicate significant Tukey-adjusted differences among species within a plant part and sampling month. Groups sharing at least one letter do not differ significantly. These means describe titer conditional on qPCR detection and do not represent all tested samples.

Monthly changes in phytoplasma titer during 2025 also differed significantly among host species (F = 1.58, *p* = 0.032). This was supported by the LRT, comparing the model with differences in monthly patterns among the examined host groups during 2025 against the simpler model (χ^2^ = 57.27, df= 33, *p* = 0.005; Table 2). Mean phytoplasma titers in roots across the 2025 sampling period ranged from 6.81 to 7.67 log_10_ cells g^−1^ phloem: *P. armeniaca* had the highest mean concentration (7.67 log_10_ cells g^−1^ phloem), followed by *P. domestica* (7.16 log_10_ cells g^−1^ phloem), *P. salicina* (6.86 log_10_ cells g^−1^ phloem) and *P. persica* (6.81 log_10_ cells g^−1^ phloem). Tukey-adjusted monthly comparisons detected no significant differences among species from January to April. From May to December, phytoplasma titer in roots of *P. armeniaca* was generally higher and, depending on the comparison, significantly higher than in the other species (Figure 2). Titers in roots of *P. salicina*, *P. domestica* and *P. persica* generally did not differ, except in October, when phytoplasma titer in roots of *P. domestica* was significantly higher than in *P. persica*.

In one-year-old shoots, phytoplasma titer differed significantly among host species (F = 12.31, *p* < 0.001) and sampling months (F = 8.22, *p* < 0.001), and the monthly pattern during 2025 differed among species (F = 4.24, *p* < 0.001; Table 2). The likelihood-ratio test further supported the differences in monthly patterns among the examined host groups during 2025 (χ^2^ = 131.38, df = 32, *p* < 0.001; Table 2). *P. domestica* had the lowest mean phytoplasma titer (6.16 log_10_ cells g^−1^ phloem), compared with 8.32 log_10_ cells g^−1^ phloem in *P. persica* and 7.98 log_10_ cells g^−1^ phloem in both *P. armeniaca* and *P. salicina*. Monthly comparisons showed that the phytoplasma titer in *P. domestica* was significantly lower than in the other three species during most of the year, specifically in February, from June to September, and in November and December. It was also significantly lower than in *P. armeniaca* and *P. persica* in April and in *P. armeniaca* and *P. salicina* in October. No positive one-year-old shoot samples of *P. domestica* were recorded in January. Finally, the three other analyzed species (*P. persica*, *P. armeniaca* and *P. salicina*) maintained high phytoplasma titers in one-year-old shoots throughout the year. In May and October, no significant differences were observed between phytoplasma titers in their one-year-old shoots (Figure 2).

In annual shoots, phytoplasma titer differed significantly among host species (F = 5.92, *p* = 0.005) and sampling months (F = 29.21, *p* < 0.001), and the monthly pattern during 2025 differed among species (F = 7.61, *p* < 0.001; Table 2). The likelihood-ratio test further supported the differences in monthly patterns among the examined host groups during 2025 (χ^2^ = 118.61, df = 17, *p* < 0.001; Table 2). *P. domestica* had the lowest mean phytoplasma titer (5.70 log_10_ cells g^−1^ phloem), based on only five positive samples, compared with 8.33 log_10_ cells g^−1^ phloem in *P. persica*, 7.97 log_10_ cells g^−1^ phloem in *P. armeniaca* and 7.69 log_10_ cells g^−1^ phloem in *P. salicina*. Monthly comparisons showed that the phytoplasma titers in annual shoots varied mostly in May and June. In May, significant differences in phytoplasma titers were recorded between *P. persica*, which had the highest titers, *P. salicina* and also *P. armeniaca*, which had the lowest titers. In June, phytoplasma titers in these three species increased; however, significant differences were observed only between *P. persica* and *P. armeniaca*. For the rest of the months (July-December), the phytoplasma titers in annual shoots of these three species were high and were not significantly different between each other. Only 5 of 96 annual-shoot samples of *P. domestica* were positive: 1 in May, 2 in June, 1 in July and 1 in October. The mean titer of these five positive samples (5.70 log_10_ cells g^−1^ phloem) is conditional on detection and should not be interpreted as representative of a typical annual shoot of this host group. Month-specific quantitative comparisons involving these five observations were, therefore, not interpreted inferentially.

### 3.3. Differences in ‘Ca. P. prunorum’ Titer among Plant Parts within Host Species

**Figure 3:**
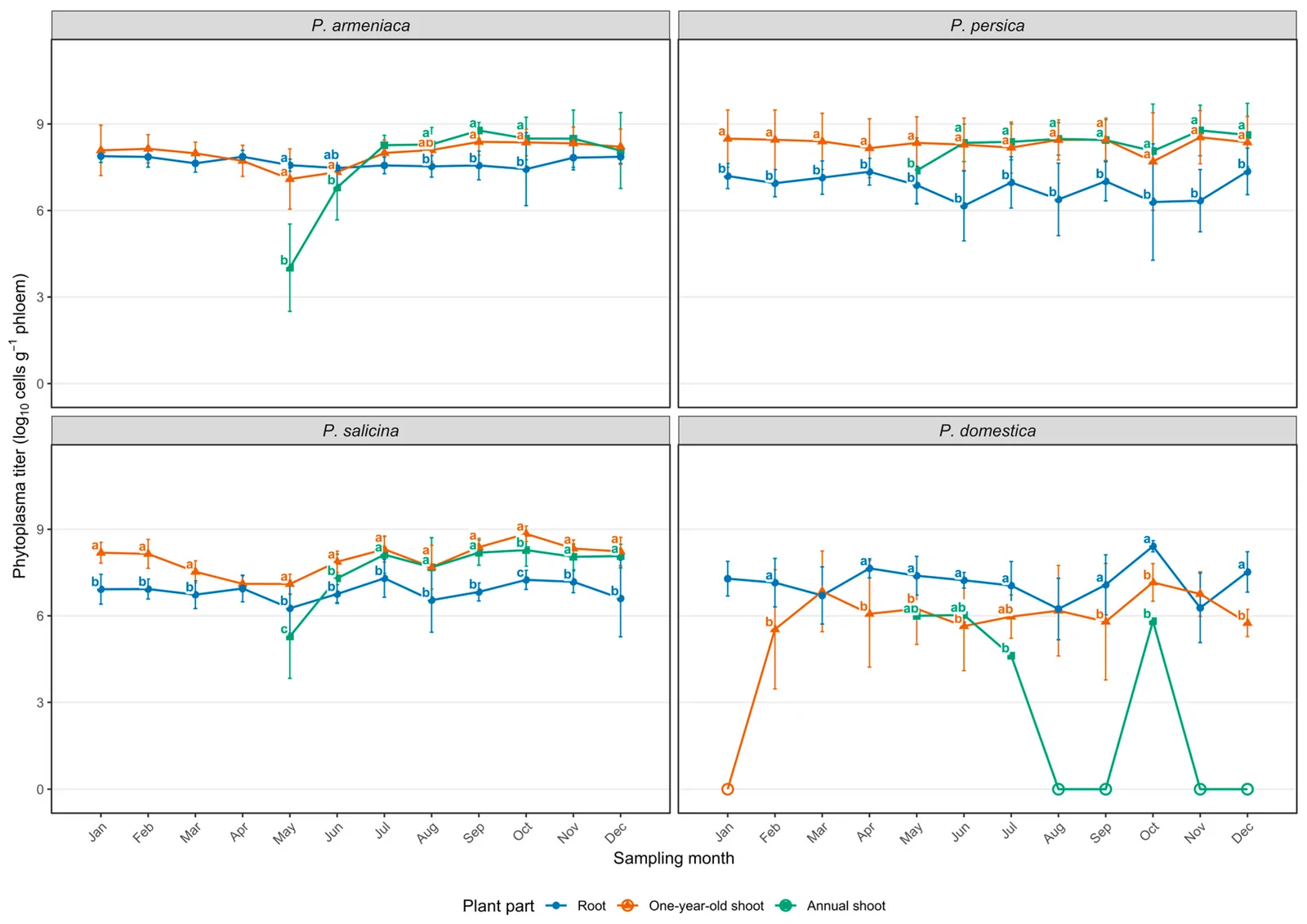
Monthly variation in phytoplasma titer during 2025 in three plant parts within individual *Prunus* species. Points represent arithmetic means calculated from positive samples, and error bars represent standard deviations (SD). Open points at zero indicate months without positive samples and were excluded from the titer models. Different lowercase letters indicate significant differences among plant parts within the same host species and sampling month after Tukey adjustment; plant parts sharing at least one letter do not differ significantly. Individual-tree phytoplasma titer trajectories for each host species, cultivar and plant parts are provided in Supplementary Figure S1. The displayed means describe titer conditional on qPCR detection and do not represent all tested samples.

Phytoplasma titer differed significantly among plant parts in all four host species: *P. armeniaca* (F = 9.12, *p* < 0.001), *P. domestica* (F = 8.94, *p* < 0.001), *P. persica* (F = 83.55, *p* < 0.001) and *P. salicina* (F = 84.49, *p* < 0.001; Table 3). The differences among plant parts changed significantly during the season in *P. armeniaca* (F = 9.76, *p* < 0.001), *P. persica* (F = 1.98, *p* = 0.020) and *P. salicina* (F = 5.92, *p* < 0.001) but not in *P. domestica* (F = 1.02, *p* = 0.440). Sampling month also significantly affected phytoplasma titer in the first three species, whereas no significant effect of sampling dates was detected in *P. domestica* (Table 3). Tukey-adjusted comparisons were subsequently used to identify the specific plant parts and months that differed significantly (Figure 3). The *P. domestica* result should be interpreted as exploratory because its annual-shoot component was based on only five positive observations.

In *P. armeniaca*, annual shoots had a lower phytoplasma titer early in the season but exceeded roots later in the season (Figure 3). Their titer was significantly lower than in roots and one-year-old shoots in May and lower than in one-year-old shoots in June. From August to October, annual shoots had a significantly higher phytoplasma titer than roots; phytoplasma titer in one-year-old shoots also significantly exceeded phytoplasma titer in roots in September and October.

In *P. persica*, phytoplasma titer in one-year-old shoots was significantly higher than in roots from January to April and in both roots and annual shoots in May (Figure 3). From June to December, phytoplasma titer in both one-year-old and annual shoots remained significantly higher than in roots, while the two above-ground plant parts did not differ significantly.

In *P. salicina*, phytoplasma titer in one-year-old shoots was significantly higher than in roots from January to March (Figure 3). In May, phytoplasma titer in one-year-old shoots was significantly higher than in roots and annual shoots, while titer in roots was significantly higher than in annual shoots. In June, titer in one-year-old shoots remained significantly higher than in both other plant parts. From July to December, phytoplasma titer in both above-ground plant parts was significantly higher than in roots. Additionally, in October, the titer in one-year-old shoots was also significantly higher than in annual shoots.

In contrast to the other host groups, *P. domestica* showed a root-dominated detection pattern (Figure 3). Roots were positive in 45 of 48 samples, compared with 57 of 144 one-year-old shoot samples and only 5 of 96 annual-shoot samples. Quantitative comparisons involving annual shoots were interpreted cautiously because only five positive observations entered the titer analysis. No positive result was obtained from one-year-old shoots in January or from annual shoots in August, September, November or December.

### 3.4. ‘Ca. P. prunorum’ Multilocus Profile Characterization and Symptom Expression

Ten ‘*Ca.* P. prunorum’ multilocus profiles were detected among the 21 analyzed trees (Table 4). Table 4 summarizes the aceF–pnp–imp–secY profiles, their distribution among the examined host species, and the number and proportion of trees carrying each profile. The most frequent multilocus profile was A8-P2-I1-S2 (five trees; 23.8%). A5-P2-I4-S2 and A6-P2-I4-S2 were each detected in four trees (19.0%), while the remaining multilocus profiles occurred in one or two trees. *P. salicina* contained the highest number of distinct multilocus profiles, whereas *P. domestica* contained only A6-P2-I10-S2 and A8-P2-I1-S2.

**Figure 4:**
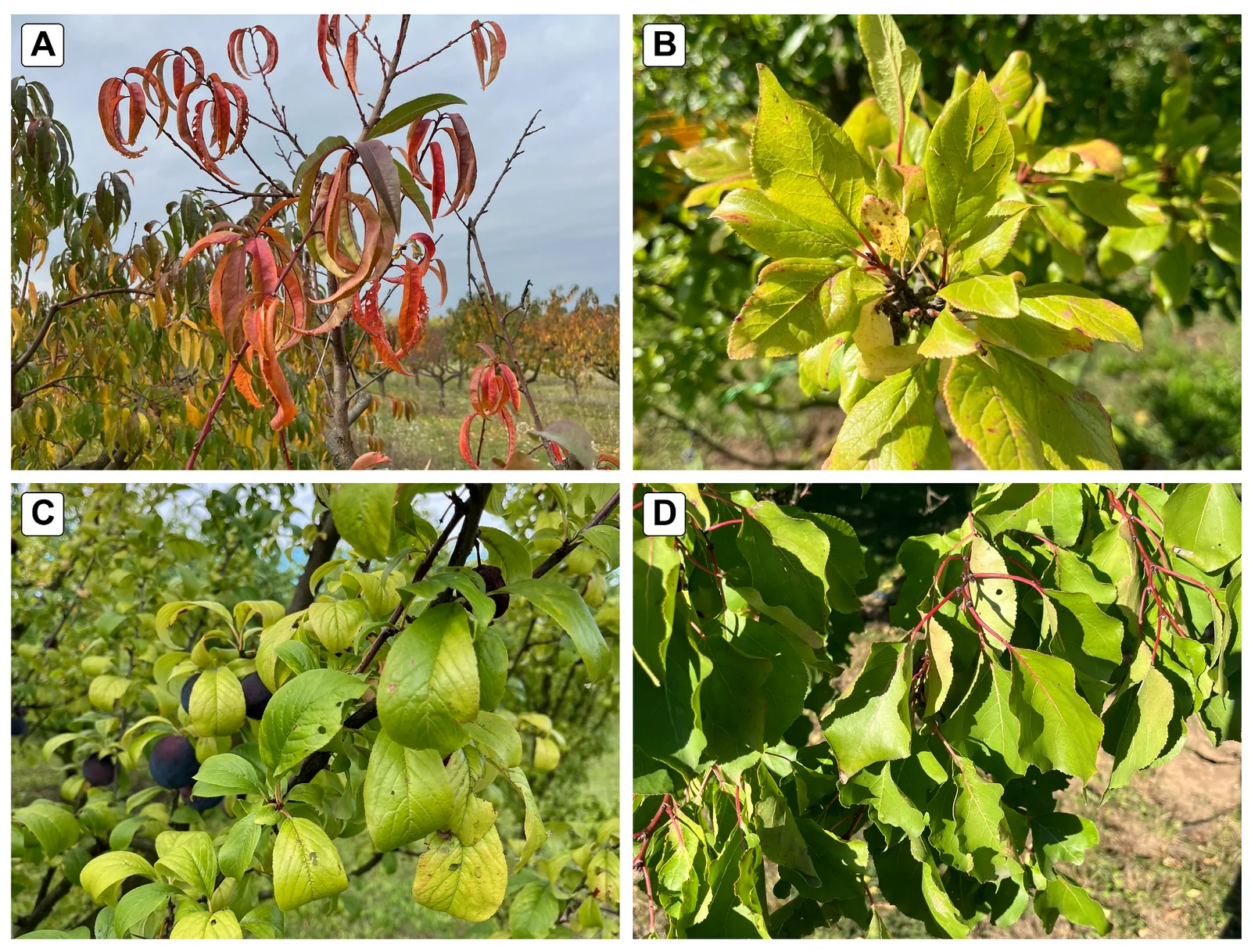
Representative foliage of monitored qPCR-positive *Prunus* trees photographed in October 2025: (**A**) pronounced leaf reddening and rolling in *P. persica*; (**B**) mild chlorosis and marginal reddening in *P. domestica*; (**C**) generalized chlorosis with mild leaf deformation in *P. salicina*; and (**D**) foliage of *P. armeniaca* with only mild leaf deformation.

During the study period, no pronounced ESFY symptoms were observed in *P. armeniaca*, *P. salicina* or *P. domestica*. Most trees of these species remained asymptomatic, with only occasional localized abnormalities. Slight leaf rolling without chlorosis was observed on one branch of the cultivar Skarb (*P. armeniaca*) (Figure 4D). Mild chlorosis without leaf rolling was recorded on some *P. salicina* trees (Figure 4C), while localized chlorosis restricted to one branch was observed in the *P. domestica* cultivar Topend (Figure 4B).

More pronounced symptoms were observed only in *P. persica*, particularly in the cultivar Helene (Figure 4A). In October, all monitored “Helene” trees showed leaf reddening and leaf rolling, followed by premature bud break in November and December. In addition, one “Helene” tree of *P. persica* with weak vegetative growth developed gummosis on the main branches and showed reduced vigor.

The more pronounced symptoms observed in the examined *P. persica* trees could be associated with the detected phytoplasma titer and may reflect a greater pathogen concentration in these trees. However, this relationship is descriptive. Given the small number of trees and the confounding of host species with cultivar, rootstock and other tree characteristics, the present data do not demonstrate that phytoplasma accumulation caused the observed differences in symptom severity. Comparison of symptom observations with the multilocus profiles did not reveal a clear multilocus profile-specific pattern. The three monitored “Helene” trees carried three different multilocus profiles, although all developed leaf reddening and leaf rolling. Moreover, A5-P2-I4-S2 was detected in both “Helene” and “Benedicte”, whereas pronounced symptoms occurred only in “Helene”. Because the multilocus profiles were unevenly distributed among only 21 trees, host species and cultivars, and most were represented in 1 or 2 trees, their relationship with symptom expression could not be evaluated reliably using statistical tests.

## 4. Discussion

This study showed that the detection rate and titer of ‘*Ca.* P. prunorum’ differed among the examined host groups, plant parts and sampling months. Across all the samples, detection was highest in roots (95.6%), followed by one-year-old shoots (87.3%) and annual shoots (76.5%). Roots, therefore, provided the most consistent detection, whereas detection in above-ground tissues varied among species and months. However, roots did not always contain the highest phytoplasma titer. Positive above-ground samples had significantly higher titers than roots in *P. armeniaca* during late summer and autumn, in *P. persica* during most of the year, and in *P. salicina* from midsummer onwards. Detection rate and titer should, therefore, be interpreted as complementary measures: detection rate describes how reliably the pathogen was detected, whereas titer describes its abundance only in samples that tested positive. Consequently, mean titers calculated only from positive samples may overestimate the overall pathogen burden in host–tissue–month groups with a substantial proportion of non-detections. The consistently high root detection agrees with previous reports that ‘*Ca.* P. prunorum’ can persist in below-ground tissues while its detectability in above-ground organs changes seasonally [2,7]; however, as shown in this study, this pattern depends on the analyzed species.

The clearest contrasting pattern among the examined host groups was observed in *P. domestica*. Roots remained frequently positive (93.8%), whereas detection decreased to 39.6% in one-year-old shoots and 5.2% in annual shoots. Among positive samples, mean titers were 7.16 log_10_ cells g^−1^ phloem in roots, 6.16 log_10_ cells g^−1^ phloem in one-year-old shoots and 5.70 log_10_ cells g^−1^ phloem in annual shoots. However, the annual-shoot mean was based on only 5 positive samples out of 96 tested and is not representative of a typical annual shoot of this host group. The quantitative model results involving this plant part should, therefore, be interpreted with particular caution. *Prunus* genotypes and rootstocks can differ substantially in ESFY susceptibility and symptom expression [2,11]. In an experimental comparison of St. Julien A, M-VA-1 and GF-305 rootstocks inoculated with two prevalent Czech haplotypes, rootstock type had a stronger overall effect on phytoplasma titer and symptom expression than the phytoplasma isolate [11]. The results presented here extend this host-dependent pattern by showing that low detection in above-ground tissues does not exclude infection when roots remain positive.

*P. armeniaca*, *P. persica* and *P. salicina* showed high overall detection rates (94.9–97.9%), but the distribution of phytoplasma titer among plant parts changed across sampling months during 2025. In *P. armeniaca*, phytoplasma titer in annual shoots was significantly lower than in roots and one-year-old shoots in May but became significantly higher than in roots from August to October. In *P. persica*, one-year-old shoots had a significantly higher titer than roots from January to May. From June to December, both one-year-old and annual shoots had significantly higher titers than roots, whereas in May, annual shoots did not differ significantly from roots. A comparable pattern occurred in *P. salicina*: one-year-old shoots had a significantly higher titer than roots early in the year, whereas both above-ground plant parts had significantly higher titers than roots from July onwards. This seasonal pattern may be consistent with the progressive colonization of newly formed shoots; however, the movement of phytoplasma within individual trees was not directly assessed. Kiss and Nečas [13] reported significantly higher phytoplasma titers in basal than in upper, younger parts of the same annual shoots during summer, a pattern that is compatible with, but does not directly demonstrate, progressive colonization of newly formed tissues.

The monthly changes observed during 2025 are also consistent with the broader biology of fruit-tree phytoplasmas. In the pear decline pathosystem, phytoplasma populations in leaves and shoots increased during spring and peaked later in the season, whereas root populations followed an approximately opposite pattern; the general seasonal course was not substantially altered by rootstock, although population levels differed among tissues and rootstock combinations [21]. Because that study involved a different phytoplasma–host system, it supports the interpretation of seasonal redistribution but does not provide direct evidence for the mechanism responsible for the patterns observed in this study.

The observed monthly changes can also be considered in relation to the seasonal physiology of phloem transport [22]. Phytoplasmas are restricted to sieve elements, and their distribution is, therefore, linked to the availability and activity of functional phloem [23]. During dormancy, cambial activity and long-distance assimilate transport may be reduced, while perennial tissues and roots can serve as storage organs [22,24]. Following bud break, cambial reactivation, the formation of new conducting phloem, and the strong sink activity of expanding shoots may provide conditions facilitating colonization and accumulation in newly developing above-ground tissues [22]. As leaves mature and become assimilate sources, and as carbon allocation changes later in the growing period, the relative transport activity of shoots, perennial tissues, and roots also changes [22]. This physiological sequence provides a plausible context for the low titer detected in young annual shoots early in their development and the subsequent increase observed in several examined host groups. The persistence of ‘*Ca.* P. prunorum’ in above-ground *Prunus* tissues during dormancy [25] indicates, however, that winter reductions in aerial colonization are not complete, unlike the stronger loss associated with the degeneration of functional aerial sieve tubes reported for some other fruit-tree phytoplasmas [26]. These interpretations remain hypotheses because cambial activity, phloem conductivity, carbohydrate allocation, and the direction of phytoplasma movement were not measured in the present study. Monthly differences in tissue titer, therefore, cannot by themselves demonstrate active redistribution. Nevertheless, the observed patterns are consistent with earlier reports of contrasting temporal changes between roots and above-ground tissues [21] and with the higher phytoplasma titer previously detected in basal than in younger upper portions of annual *Prunus* shoots [13].

In apple and pear trees, earlier anatomical and transmission studies conducted under cool-winter conditions linked the winter degeneration of functional sieve tubes with the disappearance of or marked reduction in ‘*Ca.* P. mali’ and ‘*Ca.* P. pyri’ from the aerial phloem. In most examined trees, phytoplasmas became undetectable or strongly reduced in stems during January and February, whereas roots retained functional sieve elements and remained colonized, supporting their proposed role as overwintering reservoirs [26]. Nevertheless, this pattern was not absolute, because some apple trees forming functional replacement phloem retained phytoplasmas in their stems [26]. More recent quantitative observations of ‘*Ca.* P. pyri’ also showed that the phytoplasma may remain detectable in shoots during winter, although shoot populations declined to winter minima, while root populations followed a broadly opposite seasonal pattern [21]. Rootstock influenced phytoplasma population levels but did not substantially alter the overall seasonal course [21]. By contrast, ‘*Ca.* P. prunorum’ was previously detected during dormancy in the aerial parts of several stone-fruit taxa, including apricot (*P. armeniaca*), peach (*P. persica*), Japanese plum (*P. salicina*), almond (*P. amygdalus*) and flowering cherry (*P. serrulata*), as well as in several plum rootstocks [25]. This is consistent with the year-round detection and relatively high winter titers observed in one-year-old shoots of *P. armeniaca*, *P. persica* and *P. salicina* in the present study. Thus, aerial overwintering appears to be more pronounced in the examined *Prunus* pathosystem than suggested by the classical root-reservoir model described for ‘*Ca.* P. mali’ and ‘*Ca.* P. pyri’. However, this difference should not be regarded as an absolute distinction among the three phytoplasmas because winter persistence may also be influenced by host genotype, rootstock, climatic conditions, the tissue examined and the sensitivity of the detection method. Moreover, overwintering persistence alone does not explain the differences among the examined *Prunus* hosts, particularly the low and irregular above-ground detection of ‘*Ca.* P. prunorum’ in *P. domestica*.

Additional inoculation events associated with the seasonal activity of *C. pruni* could also have contributed to phytoplasma detection in developing shoots [27,28,29,30]. However, because vector abundance, feeding sites, and transmission events were not monitored, the observed patterns cannot distinguish systemic colonization from additional vector-mediated inoculation.

A limitation of the present study is the small number of biological trees. Each host species was represented by two cultivars and two to three trees per cultivar. Although repeated monthly sampling increased the temporal resolution of the dataset, it did not increase the number of independent biological replicates. Moreover, host species, cultivar, rootstock and, partly, tree age were associated in the experimental design, and their individual effects could not be separated. The *P. armeniaca* trees were approximately 20 years old, whereas the other examined trees were approximately 10 years old. Tree age may have influenced phytoplasma distribution or symptom expression. Orchard age has previously been associated with cumulative ESFY incidence [26]. The observed differences should be interpreted as patterns among the examined species–cultivar–rootstock combinations rather than as general effects of *Prunus* species.

A further limitation is that all trees were examined at one site during a single year. The monthly patterns may have been influenced by the local climatic conditions of the 2025 season in South Moravia. Temperature and water availability can alter tree phenology, cambial and phloem activity, and seasonal source–sink relationships [22,24,31,32], which may in turn affect phytoplasma detection and titer in different tissues. Because all trees were exposed to the same local conditions and meteorological variables were not included as explanatory variables in the statistical models, their influence cannot be separated from sampling month or host group differences. Monthly mean daily temperature, mean daily maximum temperature, mean minimum temperature measured at 07:00, and total precipitation recorded in Lednice during 2025 are provided in Table S1. The observed patterns, therefore, require confirmation in multi-year studies.

The multilocus profiles detected here broadly reflect the diversity previously reported for Central European ‘*Ca.* P. prunorum’ populations. Most trees carried the P2 and S2 genotypes, and the A5-P2-I4-S2 and A6-P2-I4-S2 profiles were among the most frequent combinations, consistent with the predominance of A5/A6, I4, P2 and S2 reported in Czech and neighboring populations [11,17,18]. Less frequent profiles containing P1, S1, I10 or I13 occurred in only one or a few examined trees. These distributions may support future epidemiological comparisons among plant and insect vector populations. Ten multilocus profiles were detected among the 21 analyzed trees, but most occurred in only 1 or 2 trees and were unevenly distributed among host species and cultivars. Because only the highest-titer sample was genotyped for each tree, the assigned multilocus profile represents the dominant profile amplified in that sample and does not exclude within-tree or temporal genotype variation or mixed infection. The available data, therefore, did not allow a reliable statistical test of relationships between multilocus profile, phytoplasma titer and symptom expression. No single multilocus profile-specific symptom pattern was evident descriptively: the three “Helene” trees carried different multilocus profiles, although all developed leaf reddening and rolling, and A5-P2-I4-S2 occurred in both “Helene” and “Benedicte” although pronounced symptoms were recorded only in “Helene”. Kiss et al. [11] showed that phytoplasma genotype can influence titer and symptoms; however, this effect was not consistent throughout the experiment and was overall weaker than the effect of rootstock. The detected multilocus profiles describe variation at the four selected loci and should not be interpreted as genome-wide or phylogenetically defined lineages.

From a practical perspective, the observed tissue and monthly variation support a tiered rather than a single-tissue sampling approach. Above-ground shoots are more accessible and less invasive and are, therefore, suitable as first-line material for routine monitoring. A negative result from one shoot sample, particularly during dormancy or a period of reduced detection in the relevant host group, should be interpreted cautiously. If infection remains suspected, at least three shoots from different parts of the crown should be analyzed. Sampling should then be repeated and an alternative tissue considered if results remain negative. Roots may serve as complementary or confirmatory material because they showed comparatively consistent detection in the examined trees, but their collection is laborious, invasive and less readily standardized. Annual shoots should be sampled only after sufficient current-season growth has developed. This framework requires validation across cultivars, rootstocks, regions and years.

## 5. Conclusions

In the 21 infected trees examined at one South Moravian site during 2025, detection and positive-sample titer of ‘*Ca.* P. prunorum’ varied among sampling months and plant parts, and the observed patterns differed among the examined host groups. Sampling time and tissue type, therefore, influenced phytoplasma testing outcomes under the studied conditions. Roots provided comparatively consistent detection in this dataset, but their invasive and less readily standardized collection limits routine use; they may be considered complementary or confirmatory material when there is a high risk of false-negative results in accessible above-ground tissues. Because cultivar and rootstock were linked to host species and this study covered one site and one year, the observed differences cannot be attributed to species alone and should not be interpreted as universal diagnostic recommendations.

## Figures and Tables

**Table 1:** Plant material included in this study. SEO refers to the apricot cultivar “Stark Early Orange”.

Species	Cultivar	Rootstock	Plants (*n*)
*P. armeniaca*	SEO	Apricot seedling rootstock	3
*P. armeniaca*	Skarb	Apricot seedling rootstock	3
*P. salicina*	Angeleno	Penta^®^	3
*P. salicina*	Black Amber	Penta^®^	3
*P. persica*	Helene	Adesoto^®^	3
*P. persica*	Benedicte	Adesoto^®^	2
*P. domestica*	Stanley	Penta^®^	2
*P. domestica*	Topend	St. Julien A	2

**Table 2:** Type III F-tests evaluating the effects of host species, sampling month and their interaction on log-transformed ‘*Ca.* P. prunorum’ titer within individual plant parts. The final column presents likelihood-ratio tests comparing models with and without the host species × month interaction.

Plant Part	Host Species F-Value/*p*-Value	Month F-Value/*p*-Value	Host Species × Month F-Value/*p*-Value	Interaction LRT Χ^2^/*p*-Value
Root	5.28/0.009 **	2.28/0.012 *	1.58/0.032 *	57.27/0.005 **
One-year-old shoot	12.31/<0.001 ***	8.22/<0.001 ***	4.24/<0.001 ***	131.38/<0.001 ***
Annual shoot	5.92/0.005 **	29.21/<0.001 ***	7.61/<0.001 ***	118.61/<0.001 ***

Note: F, Type III test statistic for fixed effects in the linear mixed-effects model; denominator degrees of freedom were estimated using the Satterthwaite approximation. Annual-shoot analyses included samples collected from May to December. * *p* < 0.05; ** *p* < 0.01; *** *p* < 0.001.

**Table 3:** Type III F-tests evaluating the effects of plant part, sampling month and their interaction on log-transformed ‘*Ca.* P. prunorum’ titer within individual host species. The final column presents likelihood-ratio tests comparing models with and without the plant part × month interaction.

Host Species	Plant part F-Value/*p*-Value	Month F-Value/*p*-Value	Plant Part × Month F-Value/*p*-Value	Interaction LRT χ^2^/*p*-Value
*P. armeniaca*	9.12/<0.001 ***	24.57/<0.001 ***	9.76/<0.001 ***	119.07/<0.001 ***
*P. domestica*	8.94/<0.001 ***	1.15/0.344 ns	1.02/0.440 ns	12.37/0.261 ns
*P. persica*	83.55/<0.001 ***	2.36/0.024 *	1.98/0.020 *	28.72/0.011 *
*P. salicina*	84.49/<0.001 ***	33.31/<0.001 ***	5.92/<0.001 ***	78.70/<0.001 ***

Note: F, Type III test statistic for fixed effects in the linear mixed-effects model; denominator degrees of freedom were estimated using the Satterthwaite approximation. Analyses included positive samples collected from May to December, when all three plant parts were sampled. * *p* < 0.05; *** *p* < 0.001; ns, not significant. For *P. domestica*, interaction results should be interpreted cautiously because only five positive annual-shoot samples were available. The numbers of positive root, one-year-old-shoot and annual-shoot observations entering the models were 48, 142 and 127 for *P. armeniaca*; 30, 49 and 5 for *P. domestica*; 36, 117 and 116 for *P. persica*; and 47, 143 and 137 for *P. salicina*, respectively.

**Table 4:** Multilocus profiles detected in 21 trees. *The I4-related variant detected in the sample differed from the reference I4 genotype by a single C-to-A SNP at alignment position 196.

Multilocus Profile (aceF–pnp–imp–secY Genotypes)	Host Species (*n* Trees)	Trees (*n*)	Percentage
A3-P1-I1-S1	*P. salicina* (1)	1	4.8
A5-P2-I1-S2	*P. armeniaca* (2)	2	9.5
A5-P2-I3-S2	*P. salicina* (1)	1	4.8
A5-P2-I4-S2	*P. salicina* (2); *P. persica* (2)	4	19.0
A6-P2-I3-S2	*P. persica* (1)	1	4.8
A6-P2-I4-S2	*P. armeniaca* (3); *P. salicina* (1)	4	19.0
A6-P2-I4-related*-S2	*P. persica* (1)	1	4.8
A6-P2-I10-S2	*P. domestica* (1)	1	4.8
A8-P2-I1-S2	*P. domestica* (3); *P. salicina* (1); *P. armeniaca* (1)	5	23.8
A8-P2-I13-S3	*P. persica* (1)	1	4.8

## Data Availability

The data supporting the findings of this study are available from the corresponding author upon reasonable request.

## Supplementary Materials

The following supporting information can be downloaded at: https://www.mdpi.com/article/10.3390/microorganisms14092012/s1, Figure S1: Monthly trajectories of ‘*Candidatus* Phytoplasma prunorum’ titer in individual trees, shown separately by host species, cultivar and plant part; Table S1: Limit of detection (LOD95%) of the qPCR protocol according to Christensen et al. [14]; Table S2: Monthly meteorological conditions recorded in Lednice during 2025.
