# Supplementary material for: Seasonal Dynamics of ‘Candidatus Phytoplasma prunorum’ in Selected Prunus Species Revealed by Quantitative PCR
Source: Microorganisms. 2026 Sep 10;14(9):2012. doi: 10.3390/microorganisms14092012 (PMC13609548; doi:10.3390/microorganisms14092012)
Supplement: Supplementary file 1 [file microorganisms-14-02012-s001.zip › Figure_S1_Monthly trajectories of ‘Candidatus Phytoplasma prunorum’ titer in individual trees.pdf]

Supplementary Materials

Seasonal dynamics of ‘Candidatus Phytoplasma prunorum’ in selected *Prunus* species revealed by real-time PCR

Peter Morvay, Tomáš Kiss, Ivo Ondrášek, Erika Slámová, Eliška Zezulová and Tomáš Nečas

Figure S1 (A)

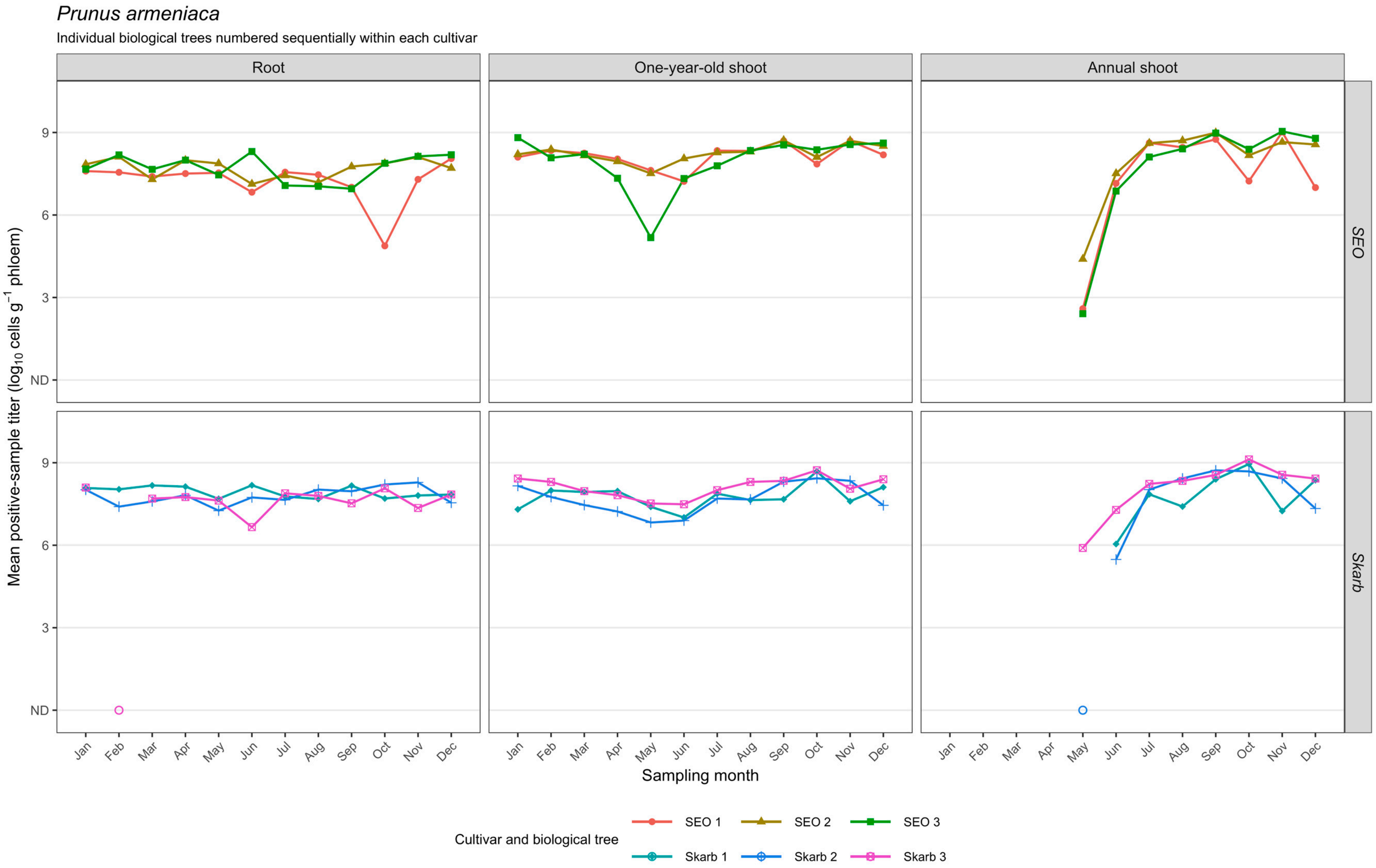

Figure S1 (A). Monthly trajectories of ‘Candidatus Phytoplasma prunorum’ titer in individual trees of *Prunus armeniaca*, shown separately by plant part. Each cultivar is shown in a separate panel row. Biological trees were renumbered sequentially within each cultivar for presentation. Points show the arithmetic mean among qPCR-positive samples for tree–month combinations with multiple tissue samples. Open symbols at ND indicate no qPCR detection and were not assigned a titer of zero. Annual shoots were sampled from May onwards. The panels are descriptive; no inferential pairwise comparisons among individual trees were performed.

Figure S1 (B)

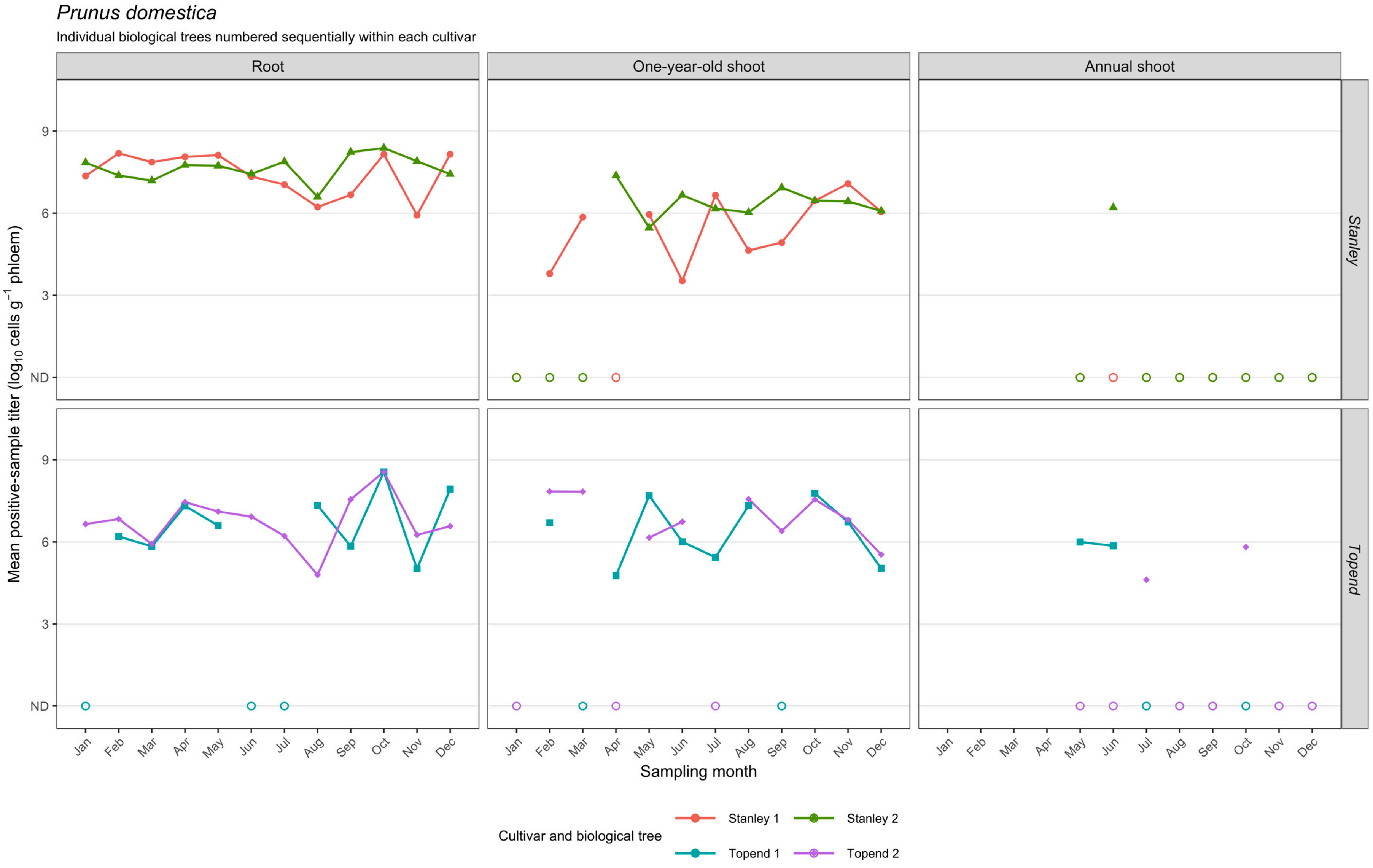

Figure S1 (B). Monthly trajectories of ‘Candidatus Phytoplasma prunorum’ titer in individual trees of *Prunus domestica*, shown separately by plant part. Plotting, aggregation, ND notation, tree-numbering conventions and interpretation are as described for panel A.

Figure S1 (C)

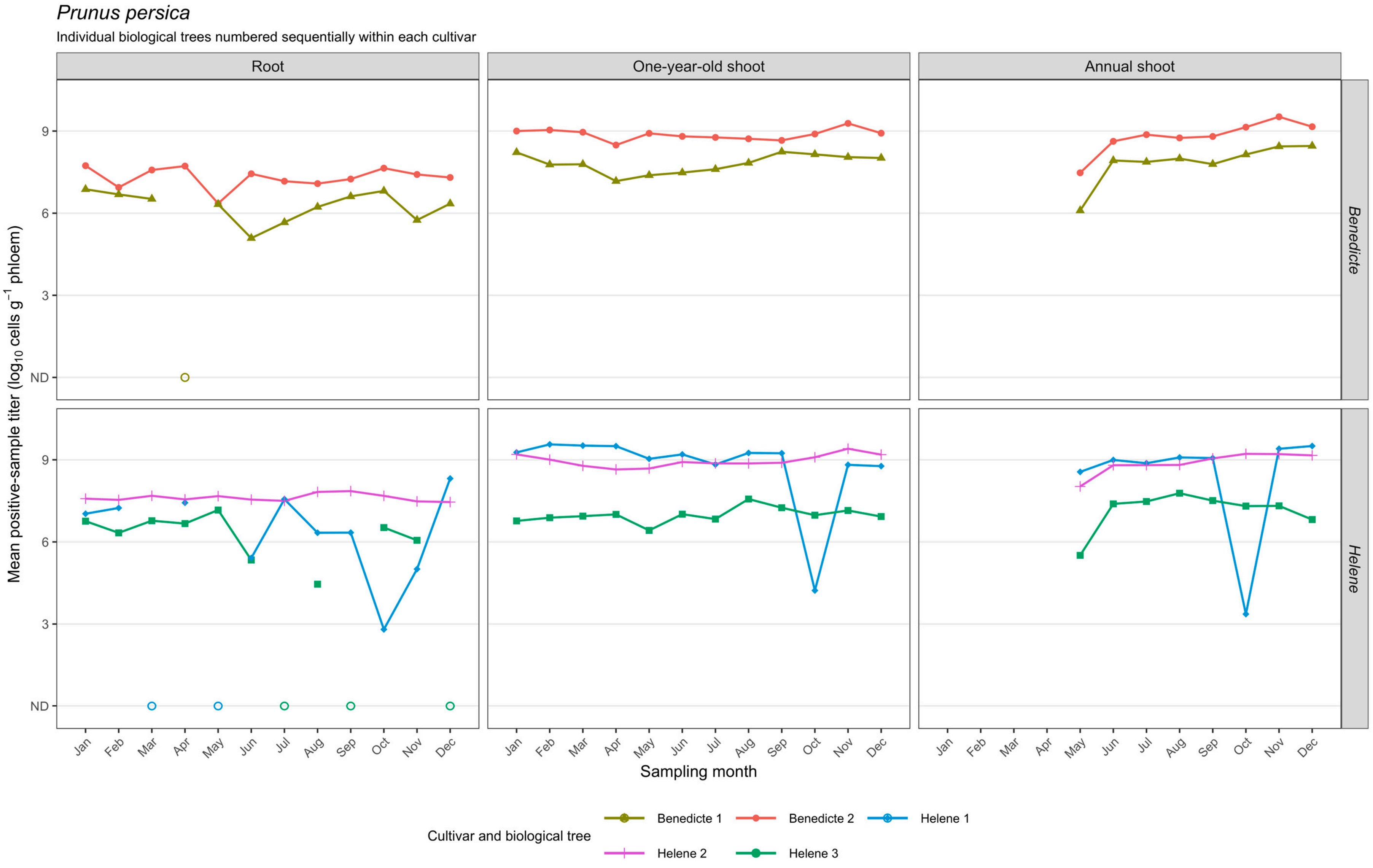

Figure S1 (C). Monthly trajectories of ‘Candidatus Phytoplasma prunorum’ titer in individual trees of *Prunus persica*, shown separately by plant part. Plotting, aggregation, ND notation, tree-numbering conventions and interpretation are as described for panel A.

Figure S1 (D)

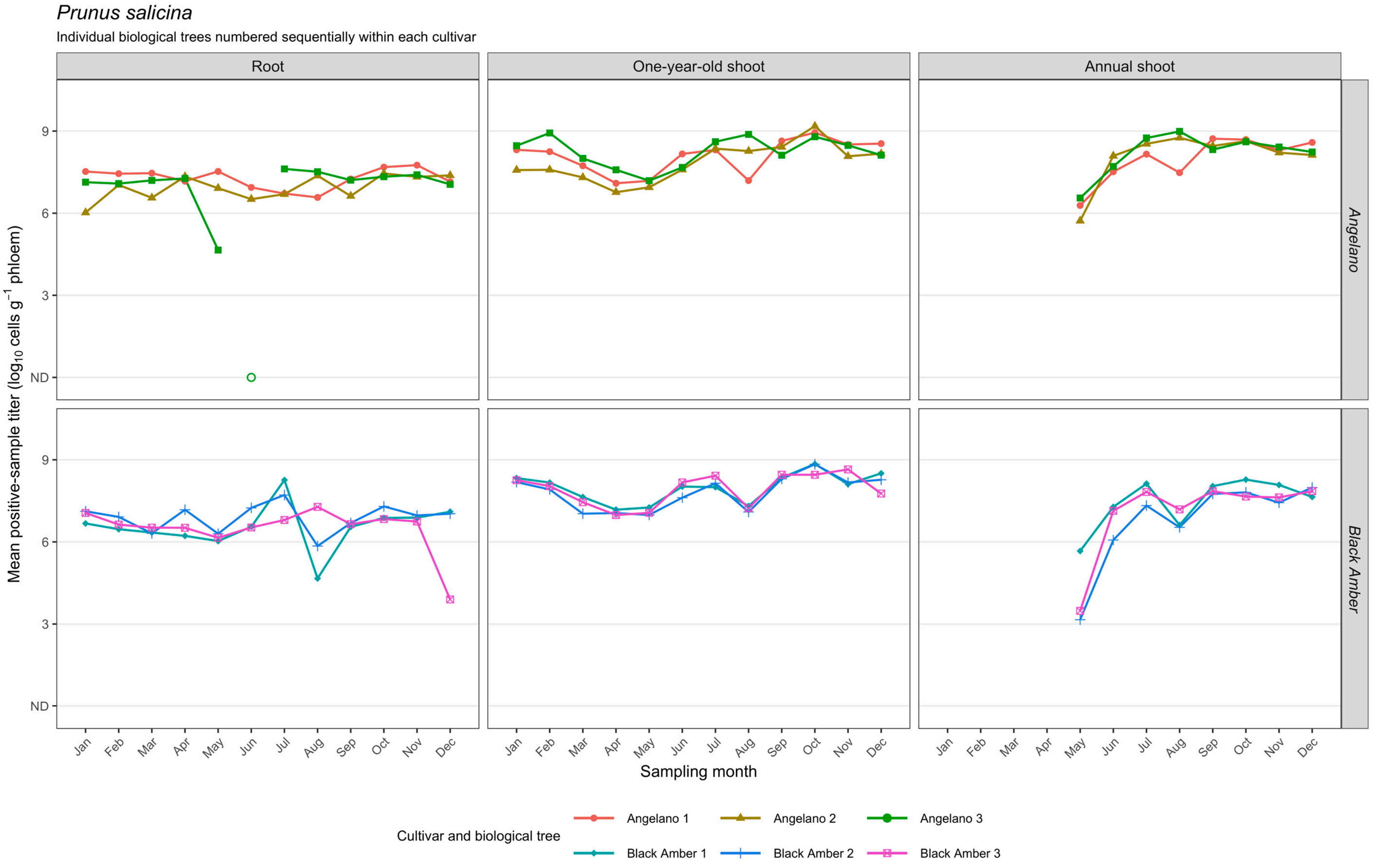

Figure S1 (D). Monthly trajectories of ‘Candidatus Phytoplasma prunorum’ titer in individual trees of *Prunus salicina*, shown separately by plant part. Plotting, aggregation, ND notation, tree-numbering conventions and interpretation are as described for panel A.
